# Inhibition of Microbiota-dependent Trimethylamine N-Oxide Production Ameliorates High Salt Diet-Induced Sympathetic Excitation and Hypertension in Rats by Attenuating Central Neuroinflammation and Oxidative Stress

**DOI:** 10.3389/fphar.2022.856914

**Published:** 2022-03-10

**Authors:** Gang Liu, Jiayin Cheng, Tianhao Zhang, Yingxin Shao, Xiangxu Chen, Lihong Han, Ru Zhou, Bin Wu

**Affiliations:** ^1^ Department of Anesthesiology, The First Hospital of China Medical University, Shenyang, China; ^2^ Department of General Practice, The First Hospital of China Medical University, Shenyang, China

**Keywords:** high salt diet, trimethylamine N-oxide, neuroinflammation, oxidative stress, hypertension, gut microbiota, dysbiosis

## Abstract

Excessive dietary salt intake induces neuroinflammation and oxidative stress in the brain, which lead to sympathetic excitation, contributing to hypertension. However, the underlying mechanisms remain elusive. Accumulating evidence reveals that trimethylamine-N-oxide (TMAO), a gut microbiota-derived metabolite, is implicated in the pathogenesis of multiple cardiovascular diseases. The present study sought to determine whether central TMAO is elevated and associated with neuroinflammation and oxidative stress in the brain after long-term high salt (HS) diet intake and, if so, whether inhibition of TMAO generation ameliorates HS-induced sympathetic excitation and hypertension. Sprague–Dawley rats were fed either a HS diet or a normal salt (NS) diet and simultaneously treated with vehicle (VEH) or 1.0% 3,3-Dimethyl-1-butanol (DMB, an inhibitor of trimethylamine formation) for 8 weeks. HS + VEH rats, compared with NS + VEH rats, had elevated TMAO in plasma and cerebrospinal fluid (CSF), increased blood pressure (BP), and increased sympathetic drive as indicated by the BP response to ganglionic blockade and plasma norepinephrine levels. HS-induced these changes were attenuated by DMB, which significantly reduced TMAO in plasma and CSF. Neuroinflammation as assessed by proinflammatory cytokine expression and NF-κB activity and microglial activity, and oxidative stress as measured by NAD(P)H oxidase subunit expression and NAD(P)H activity and reactive oxygen species (ROS) production in the hypothalamic paraventricular nucleus (PVN) were increased in HS + VEH rats but were decreased by DMB. DMB had no effects on above measured parameters in NS rats. The results suggest that long-term HS diet intake causes elevation in TMAO in the circulation and brain, which is associated with increased neuroinflammation and oxidative stress in the PVN, an important cardiovascular regulatory center. Inhibition of TMAO generation ameliorates HS-induced sympathetic excitation and hypertension by reducing neuroinflammation and oxidative stress in the PVN.

## Introduction

Hypertension is one of the most important risk factors for cardiovascular disease, which is the leading cause of mortality globally (O'Donnell et al., 2015; Grillo et al., 2019). Excess dietary salt intake has been linked to elevation in blood pressure (BP), whereas reducing dietary salt intake not only decreases the BP but also reduces morbidity and mortality from cardiovascular disease (O'Donnell et al., 2015; Grillo et al., 2019). There is considerable evidence that activation of the sympathetic nervous system plays an important role in the pathogenesis of several cardiovascular diseases, including salt-induced hypertension (Campese et al., 1982; Su et al., 2016; Liang et al., 2017; Jiang et al., 2018).

The paraventricular nucleus (PVN) of the hypothalamus is a forebrain center that integrates neural and humoral signals driving the sympathetic nervous system and plays a major role in the regulation of BP (Ferguson et al., 2008; Guyenet et al., 2020). Neuroinflammation and oxidative stress in the PVN are considered the major contributors to the augmented sympathetic nerve activity in various forms of hypertension and other cardiovascular diseases (Dange et al., 2015; Khor and Cai, 2017; Haspula and Clark, 2018; Yu et al., 2019). Excess dietary salt intake has been shown to increase neuroinflammation and oxidative stress in the PVN, which augment sympathetic nerve activity, contributing to the development and maintenance of hypertension (Liang et al., 2017; Jiang et al., 2018; Yu et al., 2019). Pharmacological interventions that inhibit neuroinflammation and oxidative stress in the PVN reduce sympathetic activation and attenuate hypertension in animals fed a high salt (HS) diet (Liang et al., 2017; Yu et al., 2019). However, the precise mechanisms by which high salt diet induces neuroinflammation and oxidative stress in the PVN remain unclear.

Emerging evidence reveals that alteration in gut microbiota, known as dysbiosis, is associated with the pathogenesis of hypertension (Marques et al., 2018; Naqvi et al., 2021). Trimethylamine N-oxide (TMAO), a gut microbial product derived from specific dietary nutrients, has been shown to be associated with cardiovascular and central nervous system disorders (Janeiro et al., 2018; Subramaniam and Fletcher, 2018; Meng et al., 2019; Yang et al., 2019). Elevated circulating TMAO induces inflammatory response and oxidative stress not only in the peripheral tissues including the heart, aorta, and kidney (Chen et al., 2017; Li et al., 2017; Li et al., 2018), but also in the central nervous system (Meng et al., 2019; Zhao et al., 2019). TMAO can rapidly cross the blood–brain barrier (BBB) (Del Rio et al., 2017). Recent studies reported that high salt intake alters gut microbiota composition and increases plasma TMAO concentration in animals (Bielinska et al., 2018; Naqvi et al., 2021).

In the present study, we examined whether central TMAO levels are elevated and associated with increased neuroinflammation and oxidative stress in the PVN of rats after long-term high salt diet intake and, if so, whether inhibition of TMAO production attenuates neuroinflammation and oxidative stress in the PVN and ameliorates high salt diet-induced sympathetic excitation and hypertension.

## Methods

### Animals

Adult male Sprague–Dawley rats at 9 weeks of age were obtained from Beijing Laboratory Animal Research Center (Beijing, China). They were housed in animal quarters with controlled temperature (23 ± 2°C) and 12:12 h light–dark cycle. The standard rat diet and tap water were provided before starting high salt diet and treatment protocols. The protocols were carried out according to the “Guiding Principles for Research Involving Animals and Human Beings”, and the experimental procedures were approved by the Institutional Animal Care and Use Committee at China Medical University (CMU2021036).

### Experimental Protocol

After 1-week acclimation, rats were randomly divided into 4 groups: 1) normal salt diet (NS, 0.5% NaCl) group treated with vehicle (VEH, tap water) (NS + VEH, *n* = 30 rats); 2) NS group treated with 1.0% 3,3-Dimethyl-1-butanol (DMB, an inhibitor of TMA formation), (NS + DMB, *n* = 30 rats); 3) high salt diet (HS, 8% NaCl) group treated with VEH (HS + VEH, *n* = 30 rats); 4) HS group treated with DMB (HS + DMB, *n* = 30 rats). Ten rats from each group were anesthetized and a telemetry transmitter was implanted in the femoral artery for continuous monitoring of arterial blood pressure (BP) and heart rate (HR). After five days recovery from surgery, BP and HR data collection was initiated. After 1-week baseline BP and HR recordings, these rats and additional 20 rats from each group without implantation of telemetry transmitters were fed a HS or a NS and treated with DMB or VEH for 8 weeks 1.0% DMB was given in drinking water and this dose of DMB has been proven to effectively inhibit TMA formation and decrease plasma TMAO concentrations in rodent (Wang et al., 2015). At the end of the experimental protocol, the ganglionic blocker hexamethonium bromide was administered (30 mg/kg, i.p.) to evaluate the sympathetic contribution to BP and HR, as previously described (Xue et al., 2011). The rats were then euthanized to collect brain, cerebrospinal fluid (CSF) and blood for molecular biochemical studies. Some rats from each group (*n* = 5/per group) were perfused transcardially with 4% paraformaldehyde for immunohistochemical study.

### Implantation of Telemetry Transmitters

Implantation of a rat BP transmitters (HD-S10, DSI, St. Paul, MN) was performed as previously described (Xue et al., 2011). Briefly, rats were anesthetized [ketamine (100 mg/kg) + xylazine (10 mg/kg) ip] and the femoral artery was accessed with a ventral incision. After the right femoral artery was isolated, the catheter of a telemetry probe was inserted into the aorta. Through the same ventral incision, a pocket along the right flank was made. The body of the transmitter was put into the pocket and secured to the abdominal wall during suture closure of the incision. After recover from the anesthesia, rats were placed in individual cages and the cages were placed on the receivers.

### Biochemical Measurements

At the termination of the experiment, rats were euthanized, and the blood was collected into prechilled 10-ml tubes containing EDTA. Plasma was separated by centrifugation for 15 min at 3000 rpm at 4°C. The levels of TMAO in plasma and CSF, the levels of norepinephrine (NE, a marker of sympathetic nerve activity), and the levels of proinflammatory cytokines IL-1β and IL-6 in plasma were determined with commercially available ELISA kits (MyBioSource, Inc. San Diego, CA, United States; Rocky Mountain Diagnostics, Inc., Colorado Springs, United States; Thermo Fisher Scientific, Grand Island, NY, United States). The levels of the advanced oxidation protein products (AOPP, a marker of systemic oxidative stress) in plasma were assessed using an OxiSelect™ AOPP Assay Kit (Cell Biolabs, Inc. San Diego, CA, United States).

### Real-Time PCR

The rats were euthanized with isoflurane and brains were quickly removed, frozen in liquid nitrogen and stored at −80°C. Coronal sections of brains were made with a cryostat microtome and bilateral PVN micropunches were taken from frozen brain sections using a rat brain slicer (Braintree Scientific Inc., Braintree, MA, United States). Total RNA was extracted from PVN tissue using RNeasy plus mini kit (QIAGEN China Co. Ltd., Shanghai, China), and cDNA was synthesized with a high-capacity cDNA reverse transcription kit (Bio-Rad Laboratories, Inc., Hercules, CA, United States). mRNA expression for the proinflammatory cytokines tumor necrosis factor (TNF)-α, interleukin (IL)-1β and IL-6, microglia marker CD11b, astrocyte marker GFAP, and NAD(P)H oxidase subunits Nox2 and Nox4, was analyzed with SYBR Green real-time PCR. The primer sequences used for real-time PCR were shown in Table 1. The ABI Prism 7000 sequence detection system (Applied Biosystems, Carlsbad, CA) was used to perform real-time PCR. mRNA data were corrected by *β*-actin and expressed as fold changes relative to the NS + VEH group.

**TABLE 1:** Sequences for primers.

Gene	Primers	Sequences
TNF-α	Forward primer	5′- AAA​TGG​GCT​CCC​TCT​CAT​CAG​TTC -3′
	Reverse primer	5′- TCT​GCT​TGG​TGG​TTT​GCT​ACG​AC -3′
IL-1β	Forward primer	5′- CAC​CTC​TCA​AGC​AGA​GCA​CAG -3′
	Reverse primer	5′- GGG​TTC​CAT​GGT​GAA​GTC​AAC -3′
IL-6	Forward primer	5′- TCC​TAC​CCC​AAC​TTC​CAA​TGC​TC -3′
	Reverse primer	5′- TTG​GAT​GGT​CTT​GGT​CCT​TAG​CC -3′
CD11b	Forward primer	5′- TGACGGCTCCGGTAGCAT -3′
	Reverse primer	5′- CCA​TCA​CAG​TTG​AGA​CAA​ATT​CCT-3′
GFAP	Forward primer	5′- GGGCGAAGAAAACCGCAT -3′
	Reverse primer	5′- TCT​GGA​GGT​TGG​AGA​AAG​TCT​GT -3′
NOX2	Forward primer	5′- CTG​CCA​GTG​TGT​CGG​AAT​CT -3′
	Reverse primer	5′- TGT​GAA​TGG​CCG​TGT​GAA​GT -3′
NOX4	Forward primer	5′- GGA​TCA​CAG​AAG​GTC​CCT​AGC -3′
	Reverse primer	5′- AGA​AGT​TCA​GGG​CGT​TCA​CC -3′
β-actin	Forward primer	5′- TGA​AGA​TCA​AGA​TCA​TTG​CTC​CTC -3′
	Reverse primer	5′- AGC​CAC​CAA​TCC​ACA​CAG​AGT -3′

TNF-α, tumor necrosis factor-α; IL-1β, interleukin-1β; CD11b, cluster of differentiation molecule 11b; GFAP, glial fibrillary acidic protein; NOX2, NADPH oxidase 2.

### Western Blot Analysis

The punched PVN tissues were homogenized in ice-cold RIPA buffer with protease inhibitor cocktail (100:1 -EMD, Millipore Corporation, MA, United States). The total protein concentration was determined by the bicinchoninic acid method (BCA Assay). Equal amounts of protein were separated by 10% SDS-PAGE and then transferred to polyvinylidene difluoride membranes (Immobilon-P, Millipore, United States). After blocking with 5% nonfat milk for 1 h at room temperature, the membrane was incubated with primary antibodies to nuclear factor kappa B (NF-κB) p65, phosphorylated (p)-NF-κB p65, NF kappa B inhibitor-α (IκB-α), and *β*-actin (Cell Signaling Technology, Danvers, MA, United States) overnight at 4°C, followed by horseradish peroxidase secondary antibodies (Santa Cruz Biotechnology, Santa Cruz, CA, United States) for 1 h at room temperature. The protein bands were visualized with an enhanced chemiluminescence kit (Amersham Biosciences, Piscataway, NJ, United States). Densitometric quantification of the bands from the blots was performed using ImageJ software (Bio-Rad, Hercules, CA).

### Assessment of NAD(P)H Oxidase Activity

NAD(P)H oxidase activity in the PVN was assessed using a NAD(P)H oxidase activity assay kit (BioVision, Milpitas, CA, United States) according to the manufacturer’s instruction. Briefly, the punched PVN tissues were homogenized in ice cold NAD(P)H oxidase assay buffer. The lysates were centrifuged at 10,000 x g for 10 min at 4°C and the supernatant was collected. Equal volumes of supernatant (50 µL) from each sample were transferred to a 96-well plate, followed by adding 50 µL reaction mix containing NAD(P)H oxidase assay buffer, NAD(P)H oxidase enzyme mix, NAD(P)H oxidase substrate I, NAD(P)H oxidase substrate II, and NAD(P)H oxidase probe. The absorbance (OD 600 nm) in kinetic mode was measured for 30 min using a spectrophotometer (Beckman Coulter, Inc., Brea, CA, United States).

### Detection of Reactive Oxygen Species

ROS production was determined in the sections at the level of the PVN by DHE staining as previously described (Liang et al., 2017; Jiang et al., 2018). Briefly, the brain was immediately frozen at −80°C for 1 h and the forebrain region containing PVN was sliced into 20-μm coronal sections with a cryostat. The sections were mounted onto glass slides, incubated in 1 mol/L DHE (Molecular Probes, Eugene, OR, United States) for 30 min at 37°C in a light-protected humidified chamber, rinsed, and imaged using a laser-scanning microscope (Zeiss LSM 710, Carl Zeiss, Inc., Oberkochen, Germany). DHE fluorescence intensity in the PVN was analyzed using NIH ImageJ.

### Immunohistochemistry

Immunohistochemical studies were performed to assess expression of Fra-like (Fra-LI, a marker of chronic neuronal activation) and CD11b in the PVN. Brains were removed from rats transcardially perfused with 4% paraformaldehyde, post-fixed in 4% paraformaldehyde for 24 h at 4°C and then cryoprotected in 30% sucrose for 48 h at 4°C. Brains were frozen in OCT compound on dry ice and sliced into 20-μm coronal sections with a cryostat. The sections were incubated for 24 h with mouse monoclonal primary antibodies against Fra-LI (Santa Cruz Biotechnology, Santa Cruz, CA, United States) or CD11b (clone OX-42, Chemicon, Temecula, United States), followed by incubations in a biotinylated anti-mouse secondary antibody (Vector Laboratories, Burlingame, United States). The number of Fra-LI positive neurons, total and activated microglia in the PVN were manually counted as previously described (Rana et al., 2010). Activated microglia were presented as a percentage of the total number of microglia.

### Statistical Analysis

All data are presented as mean ± SEM. Normal distributions and homogeneity of the variances were checked using Kolmogorov-Smirnov test and Levene’s test, respectively. Statistical analyses were performed with a two-way ANOVA followed by Tukey’s multiple comparison tests. *p* values of <0.05 were considered statistically significant.

## Results

### Effects of HS Diet and DMB on Peripheral and Central Levels of TMAO

**FIGURE 1:**
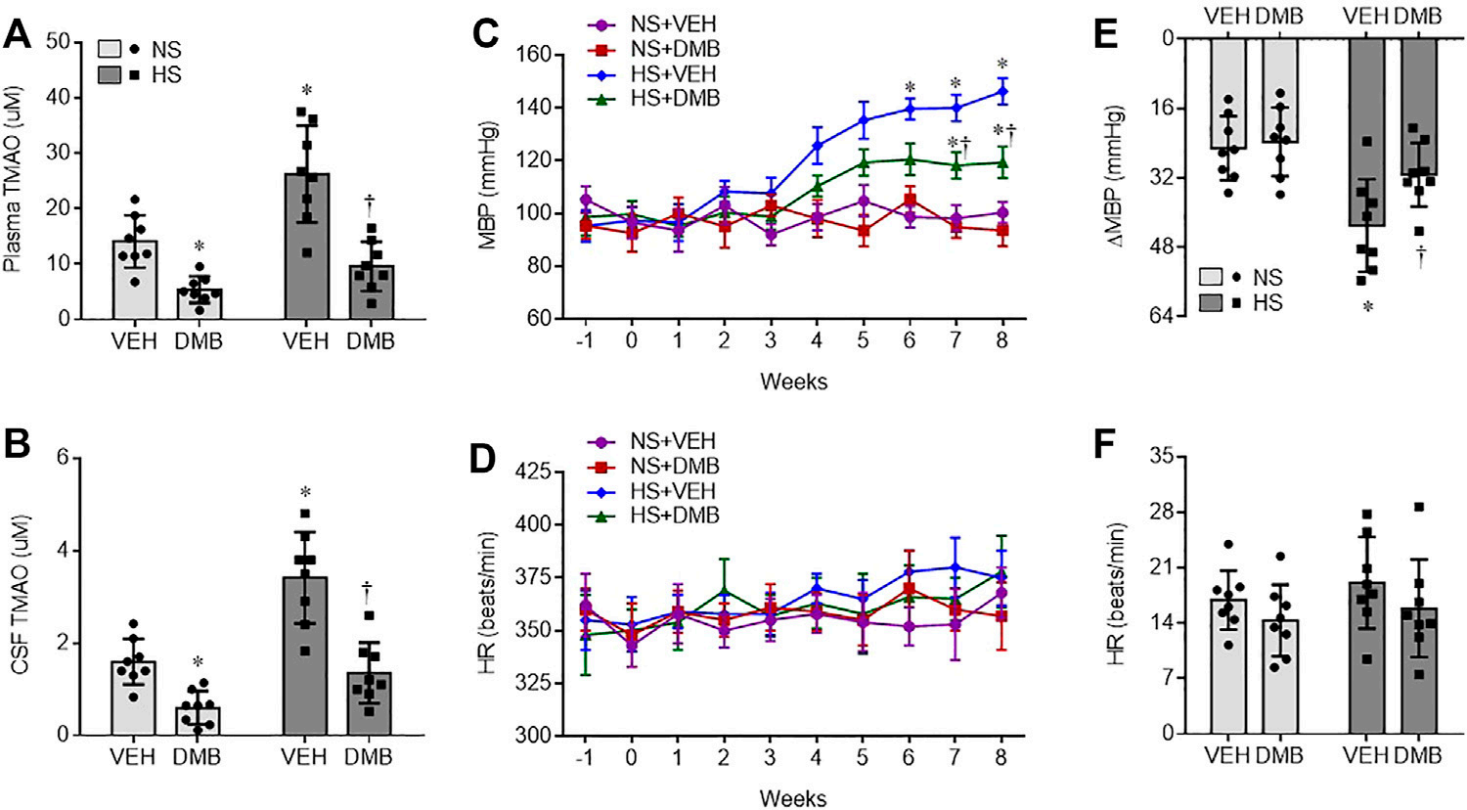
Levels of trimethylamine N-oxide (TMAO) in plasma **(A)** and cerebrospinal fluid CSF, **(B)**, and mean blood pressure MBP, **(C)** and heart rate HR, **(D)**, and maximal changes (Δ) in MBP **(E)** and HR **(F)** following administration of ganglionic blocker hexamethonium, in rats fed a normal salt (NS) diet or a high salt (HS) diet and simultaneously treated with vehicle (VEH) or 1.0% 3,3-Dimethyl-1-butanol (DMB, an inhibitor of trimethylamine formation). All data are expressed as mean ± SEM (*n* = 8 rats/per group). ∗*p* < 0.05 *vs*. NS + VEH; †*p* < 0.05, HS + DMB *vs*. HS + VEH.

At the termination of the study protocol, the levels of TMAO in both plasma (Figure 1A) and SCF (Figure 1B) were markedly higher in HS + VEH rats, compared with those in NS + VEH rats (*p* < 0.001, *p* < 0.0001, respectively). Concomitant treatment with DMB significantly reduced levels of TMAO in plasma and CSF not only in HS rats but also in NS rats, compared with respective vehicle groups (HS: *p* < 0.0001 for both plasma and CSF; NS: *p* < 0.05 for both plasma and CSF).

### Effects of HS Diet and DMB on Hypertension and Sympathetic Tone

There was no difference in baseline MBP among 4 experimental groups (Figure 1C). Compared with NS + VEH rats, HS + VEH rats experienced a gradual increase in MBP, from a baseline of 98 ± 6 mm Hg to a peak level of 145 ± 8 mm Hg at the end of week 8 (*p* < 0.0001). Concomitant treatment with DMB did not alter MBP in NS rats, but significantly prevented the increase in MBP in HS rats (*p* < 0.001). HR was comparable across groups throughout the experimental protocol (Figure 1D).

To evaluate the sympathetic contribution to BP and HR, the ganglionic blocker hexamethonium bromide was administered at the end of week 8. MBP was markedly reduced in all 4 groups in response to hexamethonium bromide (Figure 1E). Notably, the reduction in MBP was significantly greater in HS + VEH rats than in NS + VEH rats (*p* < 0.01). When compared with HS + VEH rats, HS + DMB rats had a significantly smaller depressor response (*p* < 0.05). The depressor response in NS + DMB rats was similar to that in NS + VEH rats. Hexamethonium bromide injection induced a slight increase in HR in all 4 experimental groups but no significant change in HR was observed between groups (Figure 1F).

### Effects of HS Diet and DMB on Peripheral Inflammation and Oxidative Stress

**FIGURE 2:**
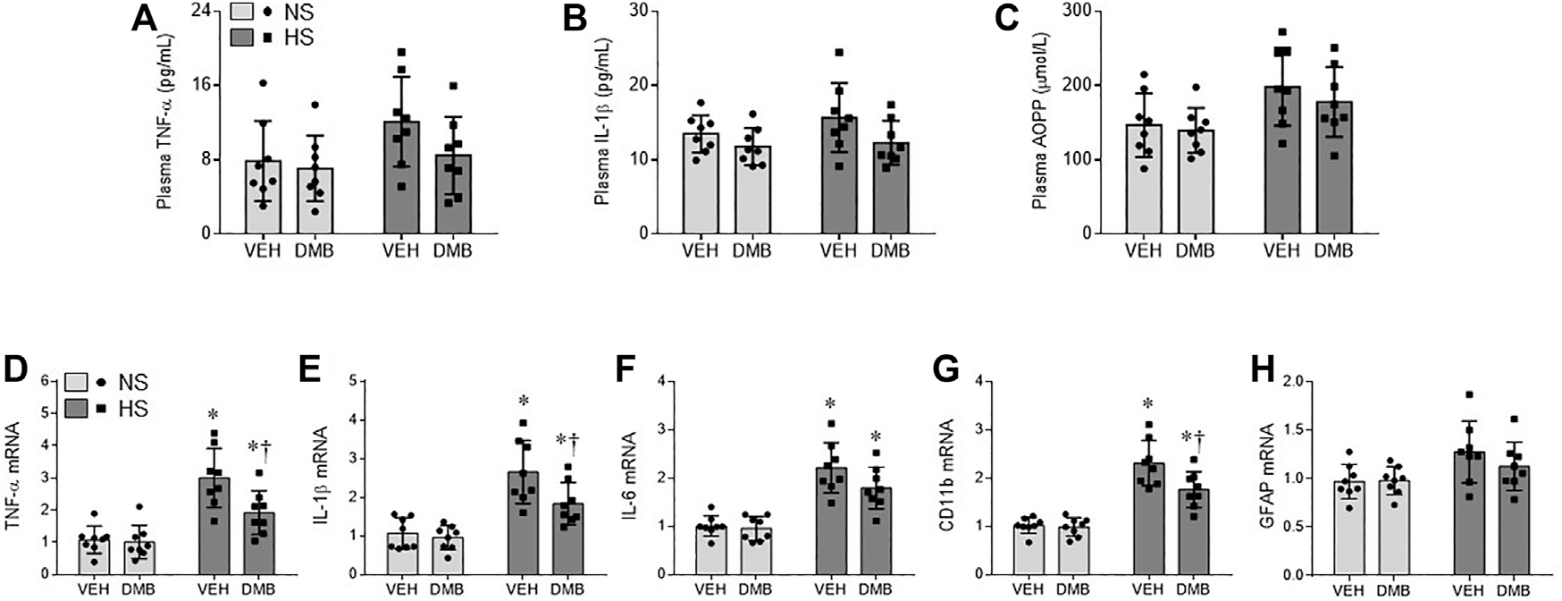
levels of proinflammatory cytokines tumor necrosis factor (TNF)-α **(A)** and interleukin (IL)-1β **(B)**, and levels of systemic oxidative stress marker advanced oxidation protein products (AOPP, **C**) in plasma, and mRNA expression of TNF-α **(D)**, IL-1β **(E)** and IL-6 **(F)**, microglia marker CD11b **(G)** and astrocyte marker GFAP **(H)** in the PVN, in rats fed a normal salt (NS) diet or a high salt (HS) diet and simultaneously treated with vehicle (VEH) or 1.0% 3,3-Dimethyl-1-butanol (DMB, an inhibitor of trimethylamine formation). All data are expressed as mean ± SEM (*n* = 8 rats/per group). ∗*p* < 0.05 *vs*. NS + VEH; †*p* < 0.05, HS + DMB *vs*. HS + VEH.

Plasma levels of inflammatory cytokines TNF-α (Figure 2A) and IL-1β (Figure 2B), and plasma levels of advanced oxidation protein products (AOPP, a marker of systemic oxidative stress, Figure 2C) in HS + VEH rats trended toward being increased compared with NS + VEH rats (*p* = 0.09; *p* = 0.25; *p* = 0.11. respectively), but the differences did not reach statistical significance. Concomitant treatment with DMB did not significantly change plasma levels of TNF-α, IL-1β or AOPP in both HS rats and NS rats.

### Effects of HS Diet and DMB on Central Inflammation

Real-time PCR showed that mRNA expression of inflammatory cytokines TNF-a (Figure 2D), IL-1β (Figure 2E), and IL-6 (Figure 2F), and mRNA expression of microglia marker CD11b (Figure 2G) were significantly increased in the PVN of HS + VEH rats compared with NS + VEH rats (*p* < 0.0001 for all). Concomitant treatment with DMB significantly reduced mRNA expression of TNF-a (*p* < 0.05), IL-1*β* (*p* < 0.05) and CD11b (*p* < 0.01) but not IL-6 in the PVN. Of note, mRNA expression of astrocyte marker glial fibrillary acidic protein (GFAP) (Figure 2H) was not different across 4 experimental groups.

**FIGURE 3:**
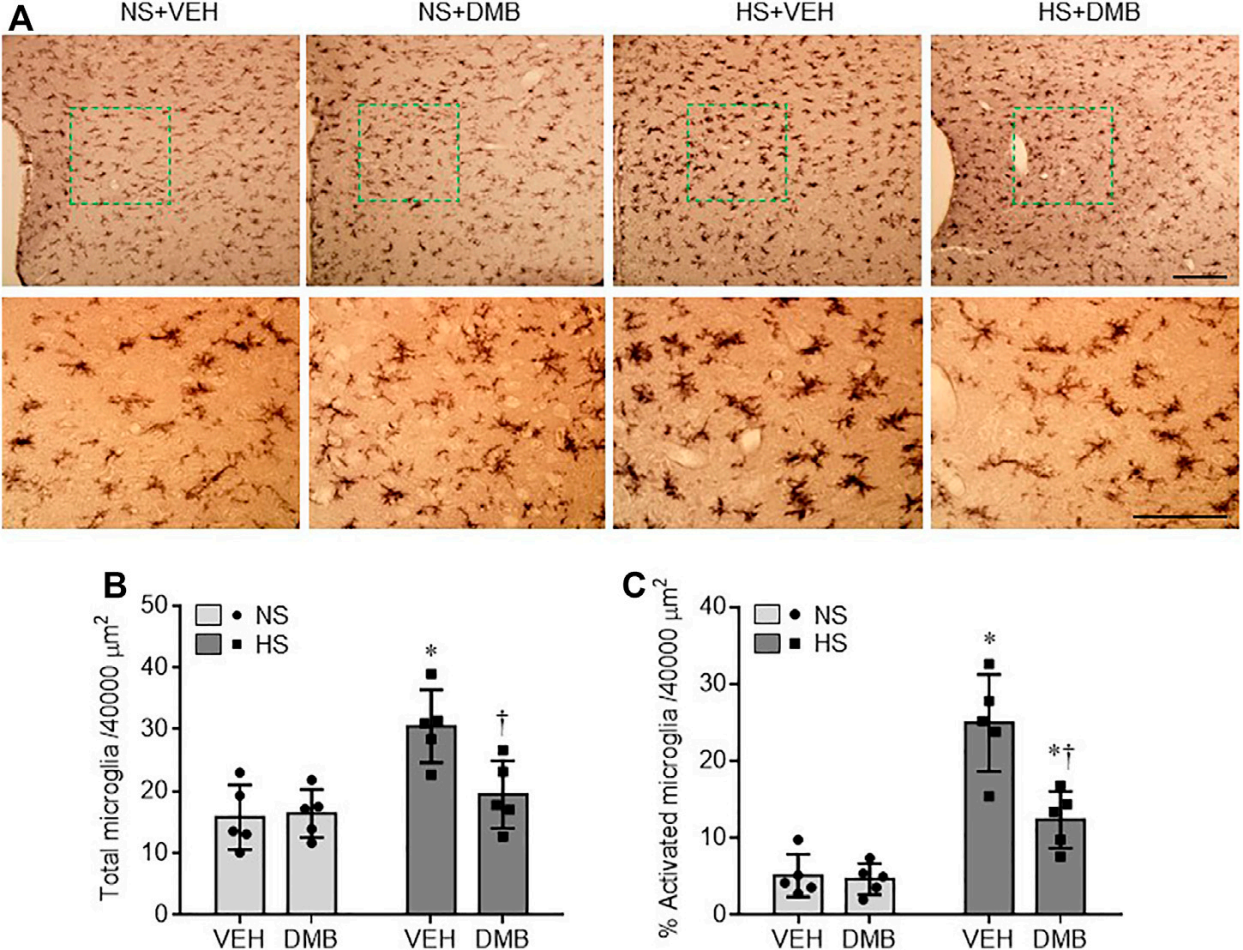
Representative images of immunohistochemistry showing CD11b-immunoreactive microglia **(A)** and quantitative comparison of total **(B)** and activated **(C)** microglia in the PVN in rats fed a normal salt (NS) diet or a high salt (HS) diet and simultaneously treated with vehicle (VEH) or 1.0% 3,3-Dimethyl-1-butanol (DMB, an inhibitor of trimethylamine formation). Scale bar = 100 μm. All data are expressed as mean ± SEM (*n* = 5 rats/per group). ∗*p* < 0.05 *vs*. NS + VEH; †*p* < 0.05, HS + DMB *vs*. HS + VEH.

Microglia has been considered the primary sources of proinflammatory cytokines in the central nervous system (Colonna and Butovsky, 2017). We therefore examined the effects of HS diet and DMB treatment on microglia activation using immunohistochemical study. As shown in Figure 3, microglia were observed in the PVN in all 4 experimental groups (Figure 3A). Compared with NS + VEH, the number of microglia in the PVN was greater in HS + VEH rats (*p* < 0.01) but was significantly lower in HS + DMB rats (*p* < 0.05) (Figures 3A,B). There are few activated microglia, as defined by strong CD11b immunoreactivity, an enlarged soma, fewer and shorter processes, in the PVN of NS + VEH rats (Figures 3A,C). In contrast, activated microglia were clearly observed in the PVN of HS + VEH rats. The number of activated microglia was significantly reduced in the PVN of HS + DMB rats when compared with HS + VEH rats (*p* < 0.001). No difference in the number of microglia or activated microglia was found between NS + DMB rats and NS + VEH rats.

**FIGURE 4:**
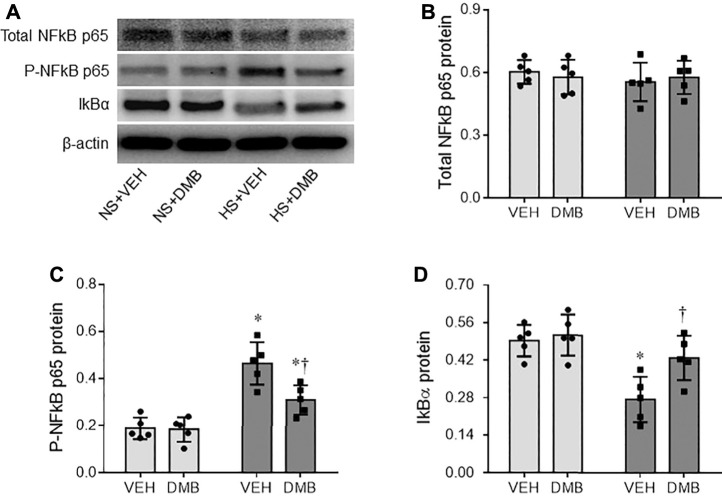
Representative Western blots **(A)** and quantitative comparison for protein levels of total NF-κB p65 **(B)**, phosphorylated (p)-NF-κB p65 **(C)** and NF kappa B inhibitor(IκB)-α **(D)** in the PVN in rats fed a normal salt (NS) diet or a high salt (HS) diet and simultaneously treated with vehicle (VEH) or 1.0% 3,3-Dimethyl-1-butanol (DMB, an inhibitor of trimethylamine formation). All data are expressed as mean ± SEM (*n* = 5 rats/per group). ∗*p* < 0.05 *vs*. NS + VEH; †*p* < 0.05, HS + DMB *vs*. HS + VEH.

NF-κB signaling pathway in the microglia plays a pivotal role in regulating production of inflammatory mediators (Dresselhaus and Meffert, 2019). We next evaluated the activity of NF-κB in the PVN. Western blot analysis revealed that the total levels of NF-κB p65 protein in the PVN were comparable among 4 groups (Figures 4A,B). However, HS + VEH rats, compared with NS + VEH rats, exhibited significant increases in the protein levels of P-NF-κB p65 (*p* < 0.0001) (Figures 4A,C) and decreases in the protein levels of NF-κB inhibitor IκB-α (*p* < 0.01) (Figures 4A,D) in the PVN. HS-induced changes in the protein levels of P-NF-κB p65 and IκB-α in the PVN were reversed by concomitant treatment with DMB, which had no effects in NS rats.

### Effects of HS Diet and DMB on Central Oxidative Stress

**FIGURE 5:**
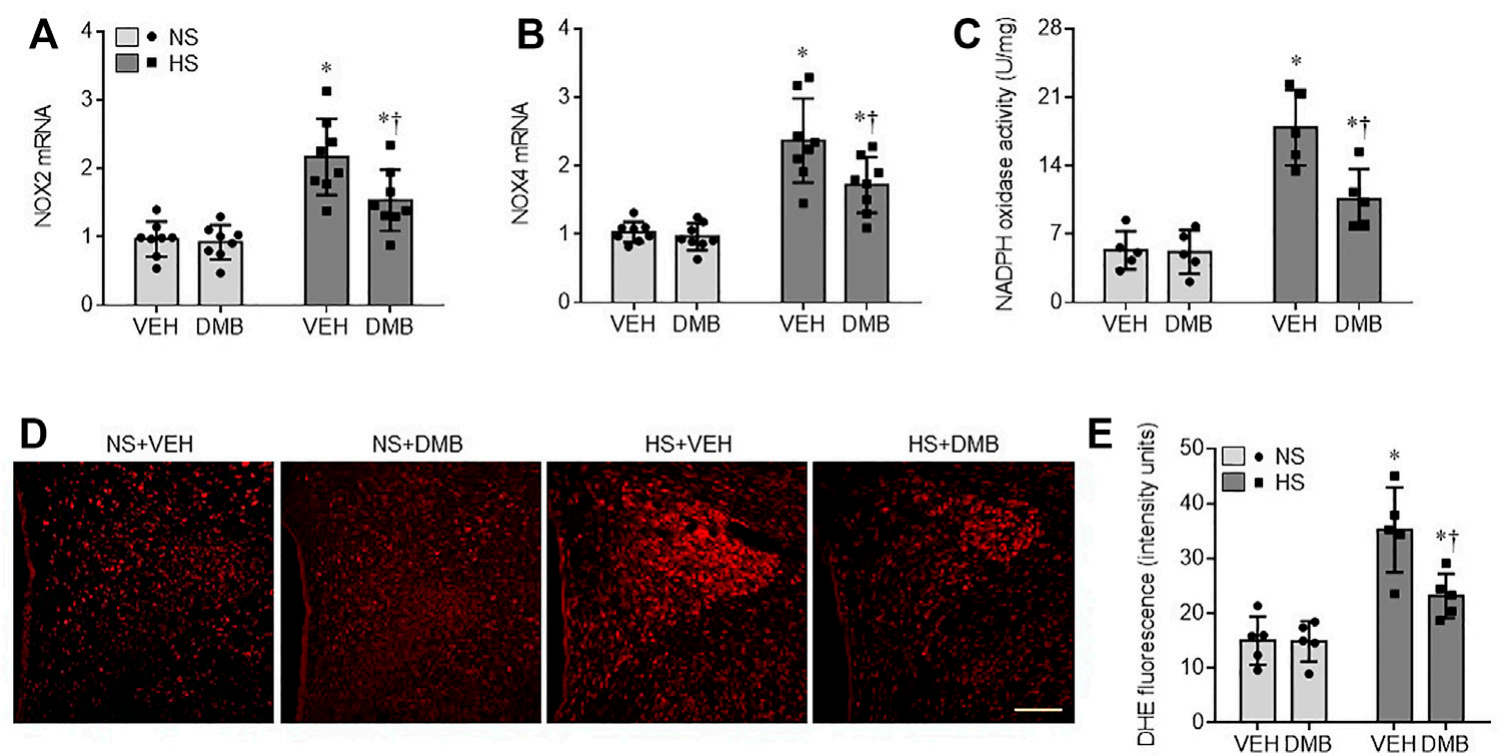
mRNA expression of NAD(P)H oxidase isoforms NOX2 **(A)** and NOX4 **(B)**, NAD(P)H oxidase activity **(C)**, and reactive oxygen species (ROS) production **(E)** as measured by DHE staining in the PVN in rats fed a normal salt (NS) diet or a high salt (HS) diet and simultaneously treated with vehicle (VEH) or 1.0% 3,3-Dimethyl-1-butanol (DMB, an inhibitor of trimethylamine formation). Representative images from each group showing DHE fluorescence are presented in **(D)**. Scale bar = 100 μm. All data are expressed as mean ± SEM (*n* = 5-8 rats/per group). ∗*p* < 0.05 *vs*. NS + VEH; †*p* < 0.05, HS + DMB *vs*. HS + VEH.

NAD(P)H oxidase-dependent ROS in the PVN has been demonstrated to be another key mediator for augmented sympathetic nerve activity and hypertension (Liang et al., 2017; Yu et al., 2019). To examine the effects of HS diet and DMB treatment on central oxidative stress, we measured mRNA expression of NAD(P)H oxidase subunits NOX2 and NOX4, NAD(P)H activity, and ROS production in the PVN. Compared with NS + VEH rats, HS + VEH rats had significant increase in mRNA expression of NOX2 (*p* < 0.0001) (Figure 5A) and NOX4 (*p* < 0.001) (Figure 5B), two major NAD(P)H oxidase isoforms expressed in the PVN, which was accompanied by elevated NAD(P)H oxidase activity (*p* < 0.0001) (Figure 5C). Moreover, intracellular ROS production detected by DHE staining, was augmented throughout the PVN in HS + VEH rats as compared to NS + VEH rats (*p* < 0.0001) (Figures 5D,E). Concomitant treatment with DMB significantly prevented the elevations of above measured parameters in HS rats but had no effects in NS rats.

### Effects of HS Diet and DMB on Activation of the Sympathetic Nervous System

**FIGURE 6:**
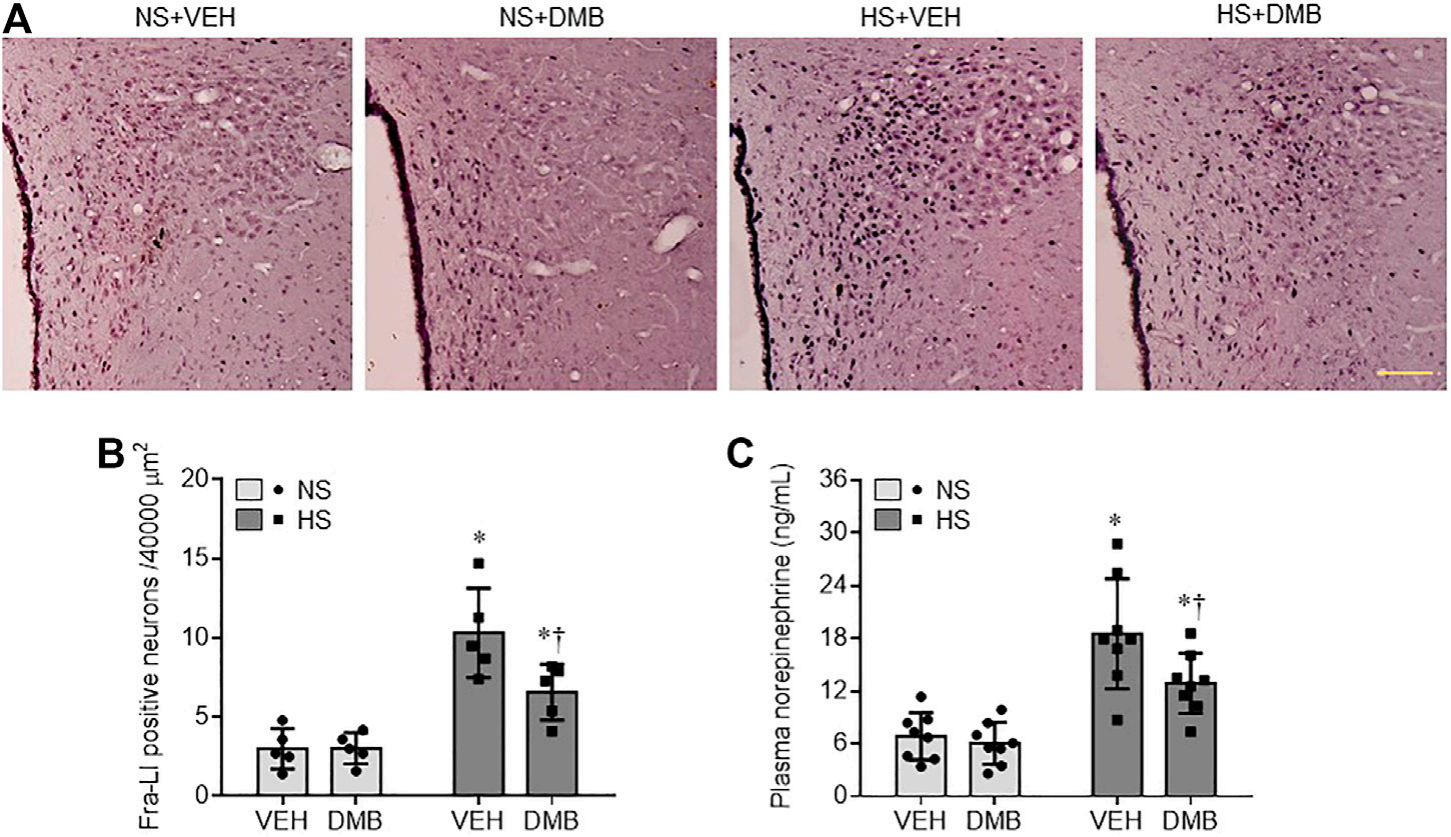
Representative images of immunohistochemistry showing Fra-LI immunoreactivity **(A)**, a marker of neuronal excitation and quantification of Fra-LI positive neurons in the PVN **(B)**, and plasma levels of norepinephrine **(C)**, a marker of sympathetic nerve activity, in rats fed a normal salt (NS) diet or a high salt (HS) diet and simultaneously treated with vehicle (VEH) or 1.0% 3,3-Dimethyl-1-butanol (DMB, an inhibitor of trimethylamine formation). Dark dots in A indicate single activated neurons. Scale bar = 100 μm. All data are expressed as mean ± SEM (*n* = 5-8 rats/per group). ∗*p* < 0.05 *vs*. NS + VEH; †*p* < 0.05, HS + DMB *vs*. HS + VEH.

We finally assessed the activation of the sympathetic nervous system by measuring Fra-LI positive neurons (a marker of chronic neuronal excitation) in the PVN, and plasma levels of NE (a marker of systemic sympathetic activity). As shown in Figure 6, the NS + VEH rats showed fewer Fra-LI positive neurons in the PVN (Figures 6A,B). The number of Fra-LI positive neurons in the PVN was markedly increased in HS + VEH rats, compared with NS + VEH rats (*p* < 0.001), but was decreased in HS + DMB rats (*p* < 0.05). Similarly, plasma levels of NE were significantly higher in HS + VEH rats than NS + VEH (*p* < 0.0001) (Figure 6C). Compared with HS + VEH rats, HS + DMB rats had reduced plasma levels of NE (*p* < 0.05). There were no differences in the number of Fra-LI positive neurons in the PVN or plasma levels of NE between NS + DMB rats and NS + VEH rats.

## Discussion

The major findings of this study are that HS feeding increases TMAO not only in the periphery but also in the brain, which is associated with elevated neuroinflammation and oxidative stress in the PVN; inhibition of TMAO production reduces neuroinflammation and oxidative stress in the PVN, ameliorates sympathetic excitation and prevents hypertension in rats fed a HS diet.

In the present study, we found that rats fed a HS diet for 8 weeks developed a stable level of hypertension. Molecular studies revealed that neuroinflammation and oxidative stress were augmented in the PVN in HS + VEH rats compared with NS + VEH rats, Moreover, blockade of ganglionic transmission with hexamethonium led to a greater depressor response in HS + VEH rats than NS + VEH rats. PVN neuronal excitation and plasma levels of NE were also significantly higher in HS + VEH rats than NS rats. These results are consistent with previous reports (Liang et al., 2017; Jiang et al., 2018; Yu et al., 2019), indicating that long-term HS diet intake causes neuroinflammation and oxidative stress in the PVN, which augment sympathetic nerve activity, leading to the development of hypertension. Importantly, we found that long-term HS diet intake increased the levels of TMAO not only in the plasma but also in the CSF. Inhibition of TMAO generation with DMB reduced the levels of TMAO in the plasma and CSF, which were associated with reductions in neuroinflammation and oxidative stress in the PVN, and improvements in sympathetic excitation and hypertension in HS-fed rats. These results extend previous findings by demonstrating that HS-induced increases in TMAO plays an important role in regulating central neuroinflammation and oxidative stress, sympathetic excitation and hypertension.

TMAO synthesis is dependent on microbial formation of TMA, which is mediated in large part by bacterial genes encoding enzymes responsible for TMA production, namely choline-TMA lyase (CutC) (Kiouptsi et al., 2018; Rath et al., 2019). TMA is absorbed from the large bowel and transferred by portal blood to the liver where the majority of TMA is oxidized to TMAO by flavin-containing monooxygenase-3 (FMO3). A previous study has shown that germ-free mice supplemented with *Clostridium* sporogenes—a CutC encoding bacterium representing only a small part of the enteric bacterial community, had 100-fold increased plasma TMAO levels, heightened platelet responsiveness and enhanced thrombosis potential when compared with germ-free mice supplemented with bacterial community that lack TMA production capacity from choline (TMA null) (Skye et al., 2018). Under normal physiologic conditions, the kidneys rapidly clear circulating TMAO *via* urinary excretion (Velasquez et al., 2016; Janeiro et al., 2018). Therefore, alterations in gut microbiota with increased CutC encoding bacterium or renal dysfunction can lead to elevated plasma TMAO levels by promoting TMAO synthesis or reducing TMAO clearance from kidney (Kitai et al., 2016; Xu et al., 2017). Experimental and clinical studies have reported that HS diet intake causes alterations in gut microbiota (Bielinska et al., 2018) and impairs renal function (Sugiura et al., 2018). Thus, increased plasma TMAO levels in HS fed rats in our study are likely due to a combination of alterations in gut microbiota with increased CutC encoding bacterium and renal dysfunction induced by a HS diet. Importantly, increased plasma TMAO levels were accompanied by elevated CSF TMAO levels, while inhibition of TMAO generation with DMB significantly decreases CSF TMAO levels. These findings are in agreement with previous study showing that circulating TMAO could cross the blood-brain barrier and might therefore be relevant to neurological function or disorders (Vogt et al., 2018).

Microglia cells are the specialized population of resident macrophages in the central nervous system responsible for immune defense. Under normal conditions, these cells are typically in a resting state, but a single stimulus or multiple stimuli exposures can induce activation of microglia (Lull and Block, 2010; Block, 2014). Activated microglia can trigger NF-κB signaling to generate multiple neurotoxic factors including proinflammatory cytokines IL-1β, TNF-α and IL-6, driving progressive neuron damage (Lull and Block, 2010; Block, 2014). In addition, activated microglia produce ROS by NAD(P)H oxidases (Lull and Block, 2010). Although ROS are beneficial to the central nervous system by damaging exogenous pathogens and play essential roles in the development of neural circuits, excessive ROS production can damage cell membranes, proteins, and DNA of neighboring neurons (Guevara et al., 2020). Moreover, excessive ROS production by microglia can also stimulate microglial generation of proinflammatory cytokines, which in turn facilitates microglial production of ROS (Guevara et al., 2020). These two components create a vicious cycle of neuroinflammation and oxidative stress enhancing each other and so resulting in neuronal dysfunction. A recent study reported that chronic supplementation with TMAO in mice induced neuroinflammation as evidenced by increased expression of proinflammatory cytokines IL-1β, TNF-α and NF-κB activity in the whole brain (Brunt et al., 2021). Additional studies showed that increased circulating TMAO exaggerated postoperative cognitive dysfunction in aged rats by activating microglia to facilitate neuroinflammation and oxidative stress in the hippocampus (Meng et al., 2019; Zhao et al., 2019). In the present study, HS + VEH rats, compared with NS + VEH rats, had significantly augmented mRNA expression of microglial marker CD11b, higher number of total and activated microglia in the PVN as measured by immunohistochemistry, which are consistent with the increases in expression of proinflammatory cytokines and NAD(P)H-dependent ROS production. DMB is a non-lethal inhibitor of TMA production by microbes and has been demonstrated to reduce circulating TMAO levels *in vivo* in rodents (Wang et al., 2015; Li et al., 2017). Our study showed that DMB treatment of HS rats markedly reduced plasma and CSF TMAO levels, which led to reductions in microglial activation, neuroinflammation and NAD(P)H-dependent ROS production in the PVN, indicating that the lowering of microglia-mediated neuroinflammation and ROS production at HS conditions is dependent on DMB action on gut microbial TMAO synthesis. Astrocytes are another type of glial cells in the central nervous system that participate in innate immune reactions and secrete inflammatory mediators. Notably, mRNA expression of GFAP, a marker of astrocyte, was comparable between HS + VEH rats and NS + VEH rats, and DMB treatment of HS rats had no effect on mRNA expression of GFAP. These results suggest that microglia but not astrocytes in the PVN play a dominant role in mediating neuroinflammation and oxidative stress in HS-induced hypertension.

The present study examined the role of TMAO on neurochemical events in the PVN that has been shown to play a major role in HS-induced hypertension (Liang et al., 2017; Jiang et al., 2018; Yu et al., 2019). However, we recognize that the effect of central TMAO on blood pressure cannot be attributed solely to neurochemical changes in the PVN. This nucleus serves only as a window on the central effect of TMAO. HS-induced increases in central TMAO levels would certainly have activated microglia in other cardiovascular regulatory centers that have been shown to modulate blood pressure, such as the subfornical organ and the rostral ventrolateral medulla (Harrison and Gongora, 2009). Further studies are warranted to determine effect of central TMAO on neuroinflammation and oxidative stress in other cardiovascular regulatory centers in HS-induced hypertension.

A limitation of this study should be acknowledged. In the present study, the activation of microglia was assessed with immunohistochemistry. Although immunohistochemistry has been widely used to measure the activation of microglia in the brain in central nervous system disorders associated with inflammation (Rana et al., 2010; Sugama et al., 2019; Han et al., 2021), it is necessary to corroborate the activation of microglia using additional methods such as flow cytometry quantification of distinct activation markers in further studies.

## Conclusion

**FIGURE 7:**
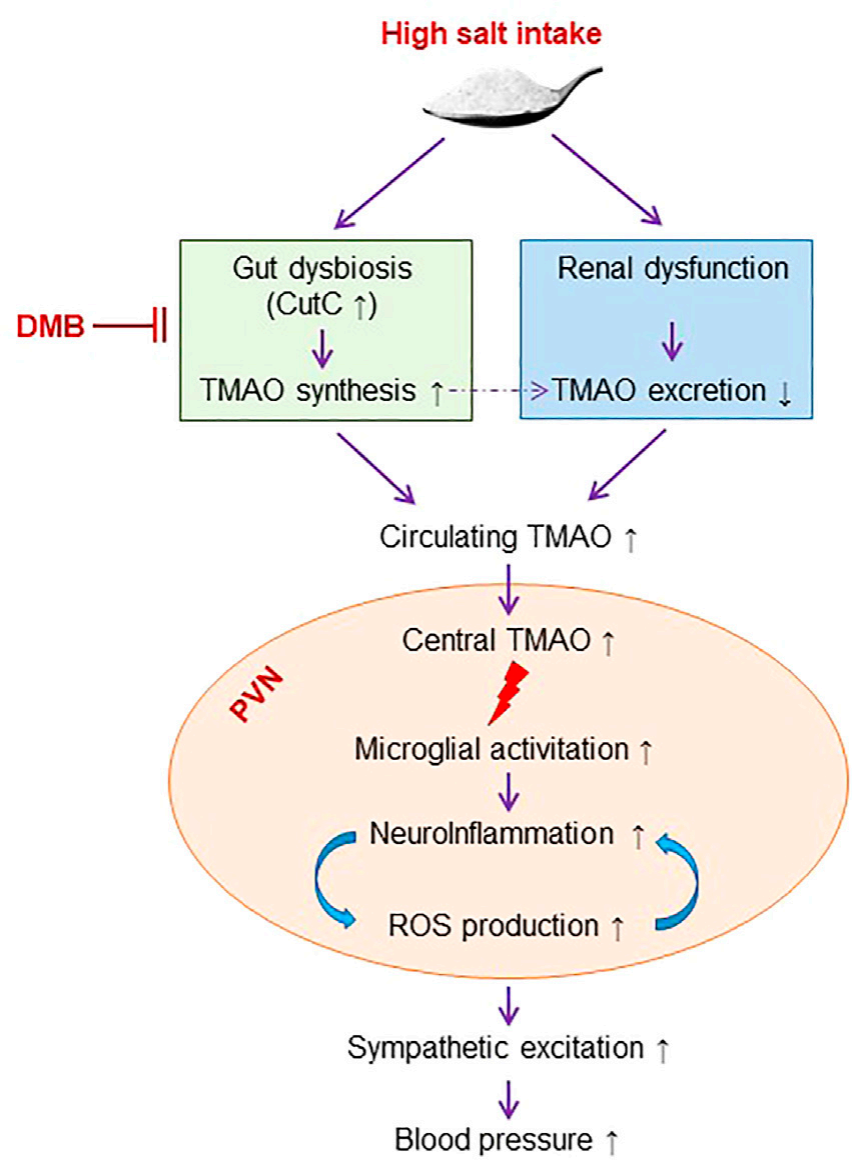
Schematic diagram showing the mechanism by which high salt intake induces sympathetic excitation and hypertension and the inhibition of TMAO synthesis for the treatment of high salt-induced hypertension. TMAO: trimethylamine-N-oxide, a gut microbiota-derived metabolite. DMB: 3,3-Dimethyl-1-butanol, an inhibitor of trimethylamine formation. ROS, reactive oxygen species. CutC, choline trimethylamine-lyase; the bacterial genes encoding enzymes responsible for trimethylamine production.

The present study demonstrates that long-term HS diet intake causes elevation in gut microbiota-generated metabolite TMAO in the circulation and brain, which is associated with increased neuroinflammation and oxidative stress in the PVN. Inhibition of TMAO generation ameliorates HS-induced sympathetic excitation and hypertension by reducing neuroinflammation and oxidative stress in the PVN (Figure 7). These findings may provide new insights into the mechanisms underlying HS-induced hypertension.

## Data Availability

The raw data supporting the conclusion of this article will be made available by the authors, without undue reservation.
